# Design of a low-cost, portable blower-based breath simulator using 3D printing for respiratory research and education

**DOI:** 10.1016/j.ohx.2025.e00731

**Published:** 2025-12-14

**Authors:** Cong Toai Truong, Trung Dat Phan, Ly Xuan Truong Pham, Huy Hung Nguyen, Tan Tien Nguyen, Van Tu Duong

**Affiliations:** a Key Laboratory of Digital Control and System Engineering (DCSELab), Faculty of Mechanical Engineering, Ho Chi Minh City University of Technology (HCMUT), 268 Ly Thuong Kiet Street, Dien Hong Ward, Ho Chi Minh City, Viet Nam; b Vietnam National University Ho Chi Minh City, Linh Xuan Ward, Ho Chi Minh City, Viet Nam; c Faculty of Engineering and Technology, Sai Gon University, Viet Nam

**Keywords:** Breathing simulator, Low-cost, Portable device, Respiration, Medical hardware, COVID-19

## Abstract

In contemporary times, as air pollution becomes increasingly severe, the challenge for healthcare in addressing respiratory-related diseases has become more urgent than ever. To assist in researching the domain of medical equipment and education training, this paper aims to create a blower-based breath simulator (BBS) for the physiological processes of spontaneous breathing by using low-cost materials and easy-to-build hardware. Specifically, the BBS focuses on providing a representation of breathing patterns, lung compliance, and airway resistance. Notably, the BBS is built on a portable 3D printable components-based structure designed for fast installation, offering direct control of breathing modes, and can be operated for a long time. Besides, the experimental test is built according to ISO 806601-2-79:2018, with testing on a dual adult training test lung from Michigan Instruments for peak inspiratory pressure, respiratory rate, positive end-expiratory pressure, tidal volume, proximal pressure, lung pressure, and demonstrating repeatability. As a result, the BBS meets initial design criteria, which comprise being lightweight, approximately 1.5 kg for the ventilator unit, and low cost, around $650 per unit, fast production time, approximately 100 continuous hours for 3D printing, and 105 h in total for the complete prototype process.

## Graphical abstract

**Figure:**
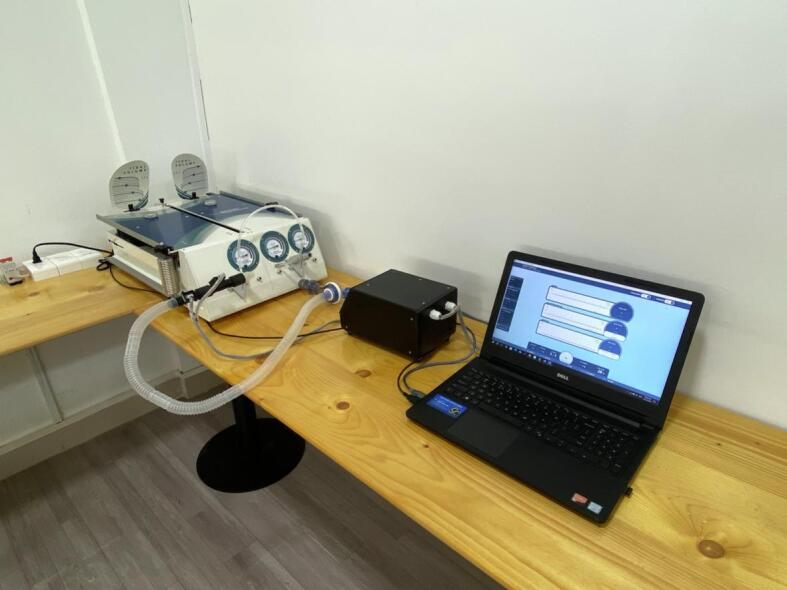

## 1 Specifications table

**Table:** 

Hardware name	Blower-based breath simulator
Subject area	• Educational tools and open-source alternatives to existing infrastructure • Medical device
Hardware type	• Breath simulation module • Breathing simulator
Closest commercial analog	ASL5000, Breath Simulation Module (Michigan Instruments)
Open-source license	CC BY 4.0
Cost of hardware	*USD 650*
Source file repository	https://doi.org/10.17632/zz3m7y3973.3

## 2 Hardware in context

Currently, respiratory care has become an issue of significant global concern, especially in light of recent events. Most notably, the COVID-19 pandemic has emphasized the top-notch importance of effective respiratory support systems in both clinical treatment and emergency response [1], [2], [3]. The COVID-19 pandemic has exposed critical shortcomings in global healthcare infrastructure, particularly the availability of mechanical ventilation [4], [5], [6]. During the early half of 2025, COVID-19 showed signs of a strong resurgence [7], with an increasing number of infections caused by new variants [8]. Hence, this has raised serious concerns about the possibility of a renewed global outbreak.

Looking back in history, the shortage of mechanical ventilators in COVID-19 was linked to increased mortality in at least 45 countries [9] in Europe alone. This crisis emphasized not only the urgent demand for affordable ventilator solutions [10], [11], [12], [13], [14], [15], [16], [17], [18], [19], [20] but also the importance of understanding and simulating the human respiratory process for training, research, and device development purposes [21], [22]. Additionally, in many developing and under-resourced countries, the ability to access, operate, and study a breathing simulator remains limited [23]. In reality, existing devices are expensive, technically complex, and inaccessible to students and researchers who lack clinical training. As a result, there is a growing need for low-cost, easy-to-use respiratory simulators that can reproduce fundamental lung dynamics for testing control algorithms, mechanical ventilators, as well as supporting medical and educational purposes.

Actually, the development of breathing simulation machines and respiratory models has been an ongoing area of research in medical and biomedical device development. Various studies have explored different approaches to replicating human respiratory mechanics, which comprise high-fidelity lung simulators, computational modeling, and mechanical ventilation test systems. First, one of the primary concerns is a high-fidelity breathing simulator. Several commercially available breathing simulators have been developed for medical training and ventilator performance assessment. Among these, the IngMar Medical ASL 5000 is a widely used high-fidelity respiratory simulator capable of reproducing complex respiratory patterns, lung compliance, and airway resistance with high precision [24], [25]. Besides, the PneuView system offers detailed real-time monitoring and control of ventilation parameters [26], [27], [28]. Alternatively, commercially available high-fidelity simulators such as the ASL 5000 (IngMar Medical) weigh over 15–20 kg and are priced in the range of tens of thousands of US dollars, while the Breath Simulation Module (BSM) − Michigan Instruments is lighter at around 1.4 kg and costs approximately 2400 USD. However, the BSM is designed as a component used in conjunction with test lungs, providing adjustable breathing patterns but lacking integrated display and standalone monitoring capabilities. These limitations, together with the high purchase price relative to academic budgets, restrict their widespread adoption in low- and middle-income countries. Furthermore, their reliance on proprietary software and specialized training limits their widespread adoption. Alternatively, several studies have focused on computational modeling to simulate human breathing dynamics. Computational fluid dynamics (CFD) models have been employed to analyze airflow distribution, lung compliance, and resistance under different physiological conditions [29], [30]. While these models provide valuable insights into respiratory mechanics, they require significant computational resources, specialized software, and expertise in biomedical engineering. Hence, computational models are limited in physically interacting with mechanical ventilators and other medical devices, making them less practical for hands-on testing and experimental validation. In recent years, portable and 3D-printable respiratory simulators have become especially popular [31], [32], [33]. Based on the recent advances in 3D printing technology, the development of portable and customizable medical devices has been enabled. Studies have demonstrated the feasibility of using 3D-printed components to create low-cost lung simulators for training and research. Nevertheless, according to international medical device regulations, these systems often lack standardized testing and validation.

From these practical challenges, this paper proposes developing a 3D-printed breathing simulation system as a dual-purpose platform. The design requirements of being lightweight and low-cost were explicitly defined to ensure accessibility and portability. A device weight of approximately 1.5–2.0 kg was targeted so that the simulator could be easily transported and deployed in classroom or laboratory environments without the need for bulky stands or external housings. A cost threshold of around 650 USD was established to make the system affordable for universities and research groups in resource-limited settings. Unlike previous efforts, the proposed blower-based breath simulator (BBS) integrates standardized physiological parameters, including lung compliance, airway resistance, and various breathing modes, allowing for a realistic representation of human respiratory mechanics. Furthermore, the BBS undergoes design based on ISO 806601-2-79:2018 standards, ensuring reliability and repeatability in its performance. Its modularity and affordability make it particularly suited for resource-limited environments where conventional systems are impractical. From there, it is a research tool for testing control algorithms and mechanical ventilation strategies, and an instructional model for universities to support hands-on learning in biomedical and control engineering programs.

## 3 Hardware description

The BBS is a compact device designed to simulate spontaneous human breathing for educational and research purposes. The hardware is constructed around a fully 3D-printed enclosure made using polylactic acid (PLA) filament, selected for its cost-effectiveness and ease of use. All mechanical parts, including the main frame, sensor brackets, blower mount, and enclosure panels, are fabricated using fused deposition modeling (FDM) technology with a total printing time of approximately 100 h. The complete BBS weighs approximately 1.5 kg and occupies a footprint of 20 cm × 16 cm × 12 cm, making it highly portable and suitable for deployment in constrained laboratory spaces or classroom settings.

The mechanical assembly is designed to ensure both ergonomic usability and structural stability. The blower is mounted horizontally using a custom 3D-printed bracket, which allows efficient directional alignment toward the outlet. Rubber feet are installed at the base to dampen vibration and provide mechanical isolation. Cable glands are incorporated at the rear panel for safe wire routing, while the front panel hosts the touchscreen HMI at a 30-degree incline for suitable visibility and operator comfort. The enclosure includes dedicated mounting slots for the flow sensor, pressure sensor, motor driver, and buzzer module, ensuring modular maintenance and replacement. Moreover, ventilation holes and internal cooling fans are included to prevent overheating during extended operation.

From the electrical perspective, the BBS is powered by a 24VDC adapter, which supplies both the high-power components and the control circuit. A buck converter steps down the voltage to 5VDC to operate the microcontroller, HMI touchscreen, and sensors integrated into the printed circuit board (PCB). A motor driver drives the blower and receives control signals from the microcontroller based on flow sensor feedback. A cooling fan is powered directly from the 24VDC and activated to ensure thermal stability. Flow and pressure sensors transmit analog signals to the microcontroller, enabling real-time measurement and adaptive control. Data is displayed on the HMI touchscreen, and firmware can be updated via a USBasp programmer through the ISP header.

Furthermore, the PCB mainboard includes built-in filtering, fuses, diodes, etc., to protect and ensure stable operation. Not over, it features extra general-purpose input/output (GPIO) and communication ports, providing expandability for additional modules, such as Wi-Fi connectivity or advanced closed-loop control systems.

As a result, the contribution of the hardware BBS is comprised of.

• Fully 3D-printed enclosure of BBS designed for low-cost and fast manufacturing.
• Compact, modular mechanical layout and portable, easy to use in various research and training applications.
• PCB mainboard with separate power routing for logic and high-power branches, integrated signal acquisition and pulse width modulation (PWM) control, compatible with open-source firmware.
• Expandable via communication and GPIO headers, enabling future research in sensor integration and ventilator control strategies.

## 4 Design files summary

The project source files and their usage are outlined in Table 1.

**Table 1:** List of components for the BBS.

Design file name	File type	Open-source license	Location of the file
Mechanical_BOM	XLSX	CC BY 4.0	https://doi.org/10.17632/zz3m7y3973.3
Electrical_BOM	XLSX	CC BY 4.0	https://doi.org/10.17632/zz3m7y3973.3
BBS_Project	PDF	CC BY 4.0	https://doi.org/10.17632/zz3m7y3973.3
BBS_Main	ZIP	CC BY 4.0	https://doi.org/10.17632/zz3m7y3973.3
Assem BBS	ZIP	CC BY 4.0	https://doi.org/10.17632/zz3m7y3973.3
Source_code_BBS	ZIP	CC BY 4.0	https://doi.org/10.17632/zz3m7y3973.3

Mechanical_BOM provides the bill for materials for all mechanical components of the BBS. It includes a complete list of 3D-printed parts, structural frames, actuator mounts, enclosures, along with detailed specifications such as dimensions, material types, quantities, and suggested manufacturing methods.

Electrical_BOM: contains the bill of materials for the electronic circuit of the BBS. It lists all necessary electrical components, including resistors, capacitors, ICs, connectors, and power devices, with corresponding part numbers, values, quantities, supplier references, and ordering links.

BBS_mainboard: comprises the schematic design and PCB layout of the main control board used in the BBS. It specifies component interconnections, signal routing, and power distribution, supporting system control and peripheral communication.

BBS_Main: includes the complete Altium Designer project, encompassing the main BBS controller's schematic and multi-layer PCB layout. It defines the functional structure of the board, component placements, track routing, and layer configuration, ensuring signal integrity and electrical performance during operation.

Assem_BBS: contains the 3D mechanical assembly model of the BBS, integrating the PCB, mechanical enclosure, actuator mechanisms, HMI, and components. It ensures proper alignment, structural compatibility, and clear visualization for assembling the entire system.

Source_code_BBS: includes the firmware for the BBS controller, implementing all control algorithms for breathing simulation. It handles configuration of breathing parameters (e.g., tidal volume, BPM, I: E ratio), actuator control, and communication.

## 5 Bill of materials summary

In this section, the bill of electrical and mechanical materials is included in supplementary materials to make it easy for the reader to get in touch with them, as they consist of many parts and components. Specifically, the bill of electrical materials is comprehensively provided in the supplementary materials and outlines all components required for assembling the PCB mainboard. Explicitly, the part numbers, quantities, component values, footprints, and manufacturer references are listed in detail in this document. To maintain completeness, the list has been exported directly from the Altium Designer project for consistency between schematic and physical layout. Components in the list include microcontrollers, communication modules, resistors, capacitors, and connectors. This is going to be used as a complete reference during procurement, fabrication, and quality assurance for PCB mainboard assembly, more so in an academic or prototyping environment. Moreover, the mechanical bill of materials is included in the supplementary materials, which consists of everything needed for the physical assembly of the BBS. This includes the 3D-printed parts for the frame, actuator mounts, enclosure panels, and structural supports, all designed in SolidWorks for FDM printing with PLA or recycled plastic filaments. Alongside that, off-the-shelf components, which comprise the screen, cooling fan units, screws, bolts, and spacers, are listed with specific dimensions and specifications. Each part is annotated with its quantity, material type, and recommended sourcing options, facilitating straightforward assembly and procurement for research or educational deployment.

## 6 Build instructions

### 6.1 Overall system description

**Fig. 1:**
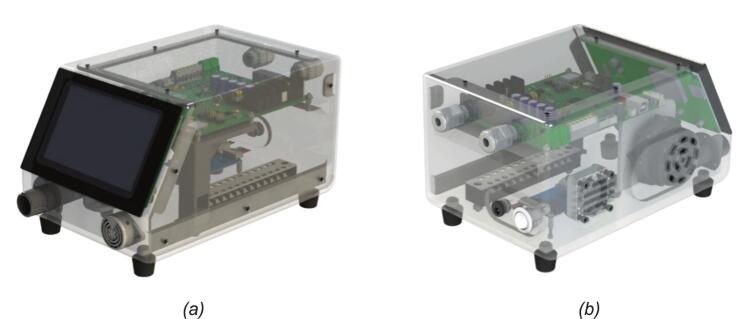
The BBS transparent 3D model: (a) transparent front view; (b) transparent rear view.

Fig. 1 presents a transparent 3D model of the BBS, showcasing the internal arrangement of key components within the BBS’s enclosure. The transparent casing reveals a well-organized internal structure with various component arrangements. The blower mounted on the left side is prominently visible and is responsible for generating the airflow required to simulate human breathing. Airflow is directed through internal channels and connectors, with visible ports for tubing connections on the front and side panels. At the top, a PCB mainboard is mounted horizontally, integrating the core electronic components. It handles control and sensor signals, as well as BBS’s parameters from operator input. And below, the driver motor is placed near the blower to control it conveniently. Additionally, the terminal blocks are designed to organize and route the wiring. Power and communication cables are neatly routed through dedicated cable glands for safety and organization on the rear. On the front, the HMI touchscreen is placed on the inclined surface to create an ergonomic, easy-to-use interface with the operator. Simultaneously, a buzzer module is integrated to provide warnings in hazardous or critical situations.

### 6.2 Installation guide

**Fig. 2:**
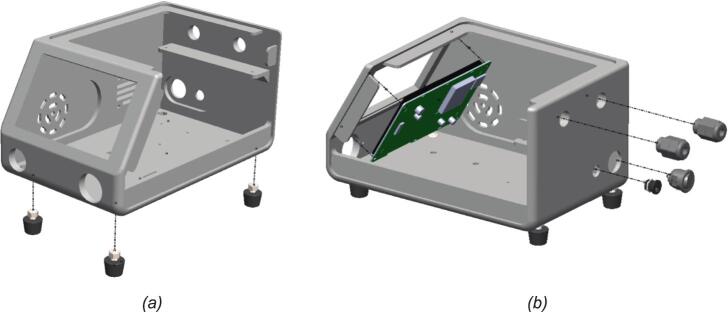
Installation of rubber feet, HMI touchscreen, and cable glands for BBS: (a) mounting the rubber feet onto the enclosure base; (b) installation of HMI touchscreen and cable glands.

Fig. 2 shows the installed rubber feet, HMI touch screen, power jack, and cable glands for BBS. The assembly begins with installing four rubber feet on the base of the main enclosure. These feet are essential for mechanical stability and vibration isolation. Rubber feet are fastened using a screw inserted through pre-drilled holes in the chassis, as shown in Fig. 2a. This step ensures a stable platform for subsequent component integration and prevents direct contact between the housing and the working surface, allowing better air circulation. In Fig. 2b, the HMI touchscreen is mounted onto the front face of the enclosure and secured using mounting screws. It serves as the primary operator interface for real-time control and parameter input. On the side wall, PG9 cable glands are installed through circular cutouts to facilitate sealed cable routing. These glands allow sensors and actuator wires to pass into the housing while protecting internal electronics from dust and moisture ingress.

**Fig. 3:**
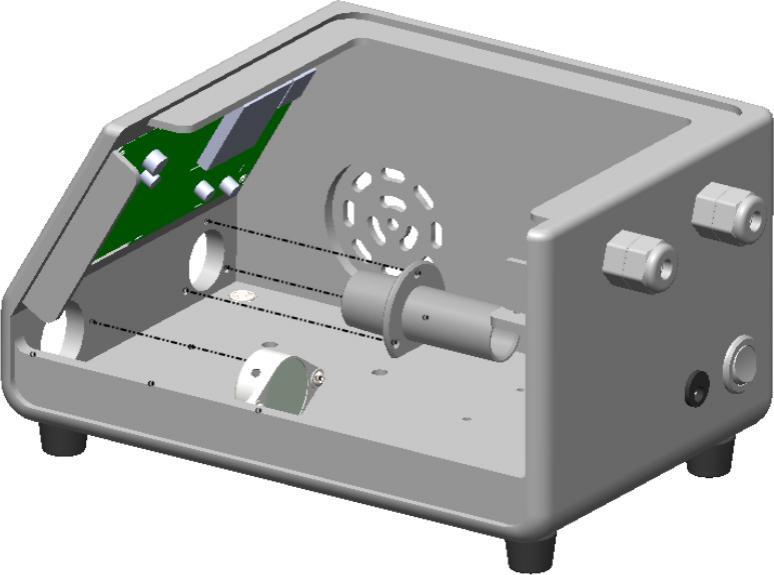
Integration of the blower outlet and buzzer module.

In this assembly step, the air blower-output connector, as shown in Fig. 3, is mounted onto the enclosure to transfer with the airflow generated by the internal blower. The connector is aligned with the machining circular opening on the front enclosure and secured to ensure no displacement during the operation of BBS. This fitting serves as the primary outlet through which the simulated airflow is delivered to external equipment such as the mechanical ventilator or the spontaneous breathing lung. Simultaneously, a buzzer module fits into a dedicated mounting slot on the enclosure. This module is an audible alarm to notify operators of abnormal conditions such as overpressure, disconnection, or sensor failure. Both the blower-output connector and the buzzer module are fixed using screws and press-fit mechanisms to ensure mechanical stability throughout continuous operation.

**Fig. 4:**
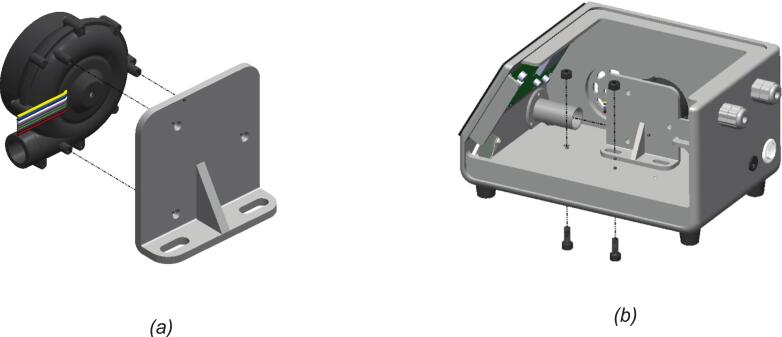
The blower installation bracket assembly: (a) blower and mounting-bracket subassembly; (b) installation of the blower–bracket assembly inside the enclosure.

Before the blower-bracket assembly is mounted in the enclosure, as shown in Fig. 4b, the next step is to mount it on a bracket assembly first, as shown in Fig. 4a. The bracket provides structural support and flexibly aligns the position of the blower within the enclosure. These components are pre-assembled with each other before being installed into the BBS. After putting it in the position, the bracket alignment and sliding fit are critical to guarantee an airtight coupling, which is essential to prevent leakage and maintain stable pressure delivery. Alongside that, the blower and bracket subassembly are secured in BBS by bolts and nuts. This step positions the blower wires close to the controller for efficient wiring.

**Fig. 5:**
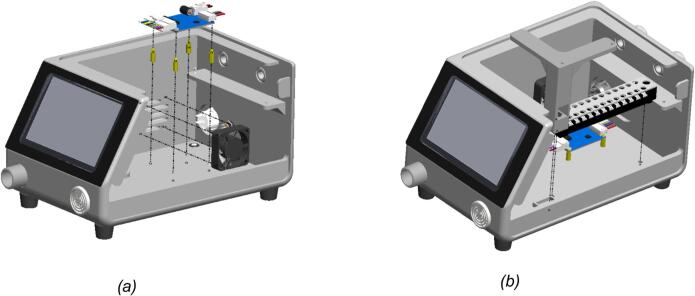
Fitting the fan, driver, and terminal wire: (a) installation of the cooling fan and motor driver; (b) installation of the terminal block and internal support bracket.

Fig. 5a illustrates the cooling fan and motor driver mounted in the BBS. A cooling fan is installed adjacent to the control zone to prevent overheating during extended operation. The fan is connected to the internal circuit using a custom cable harness. All cables are routed vertically and organized through wire management paths to minimize electromagnetic interference and physical obstruction. Additionally, a 3D-printed support bracket is installed from above to the base of the enclosure. It is responsible for holding the PCB mainboard (in the next step) securely in position, as depicted in Fig. 5b. This support bracket is fastened using screws, providing mechanical rigidity and protection against vibration-induced misalignment during operation.

**Fig. 6:**
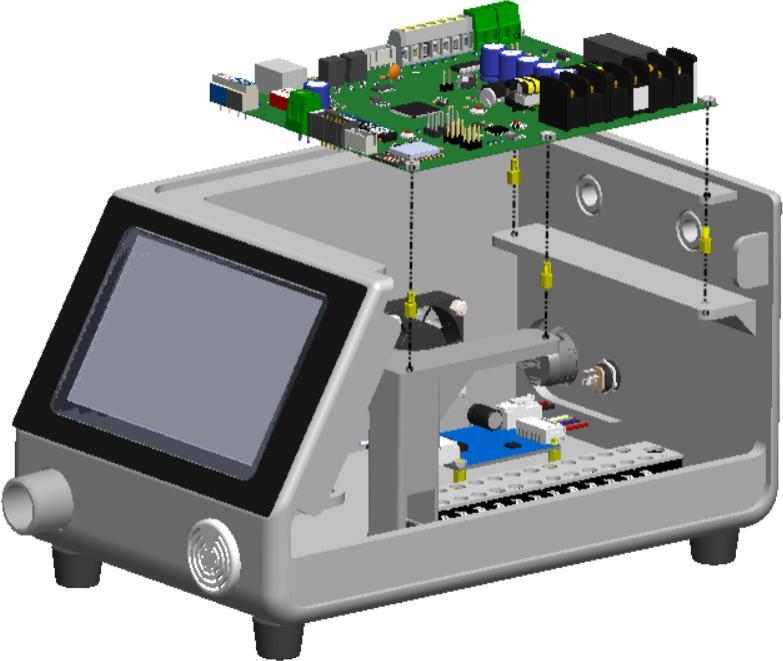
Mounting the PCB mainboard with insulation support.

In the penultimate step, the PCB mainboard installation is conducted and shown in Fig. 6. Whereby, the main control PCB is assembled at the top of the enclosure using brass standoffs and screws for elevation and insulation. This board houses the microcontroller, control signal motor driver, ADCs, signal circuits, etc. Especially, the PCB mainboard setup is vertical, which aids heat dissipation and easy component accessibility.

**Fig. 7:**
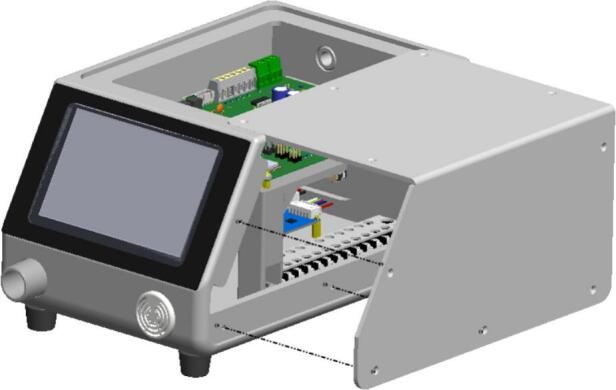
Final assembly of the top cover enclosure.

And the last step, Fig. 7 depicts the direction of the final enclosure assembly of the BBS. The panel is aligned and secured with screws to complete the whole cover. It serves several critical functions in the overall design of the BBS. In terms of advantage, it provides mechanical protection, shielding internal components such as the PCB mainboard, blower, and driver motor from external impacts, dust, and moisture. It ensures electrical safety as it is made of plastic, preventing accidental electrical contact, thereby reducing the risk of short circuits or electric shock. From a usability standpoint, the enclosure enhances the BSS’s aesthetic appearance, conceals wiring, and contributes to a compact, professional form factor suitable for laboratory or educational use.

### 6.3 Internal wiring and component integration

**Fig. 8:**
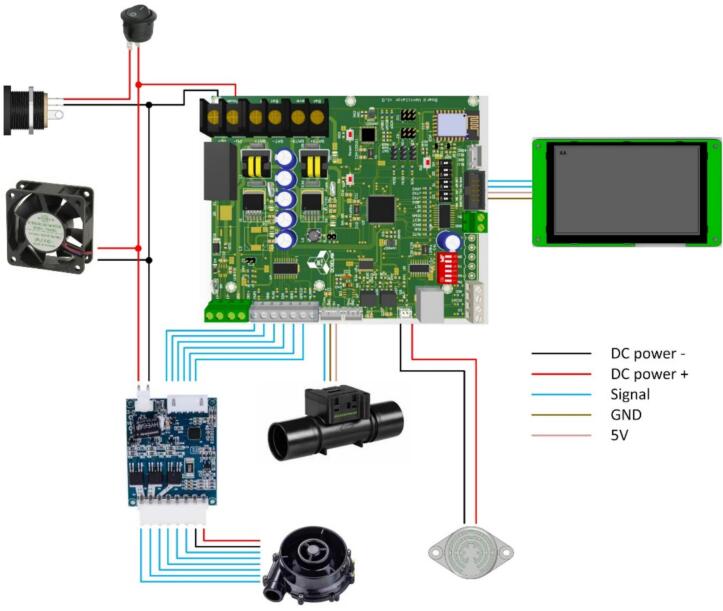
Diagrams of interior wiring and components of the BBS.

Fig. 8 presents the internal wiring and component integration of the BBS. Initially, the 24VDC power supply is connected to the main controller board through the input terminal, providing primary power to the entire BBS. Once energized, the buck converter steps down from 24VDC to 5VDC for low-power peripherals, which comprise sensors, a microcontroller, and the HMI touchscreen. Regarding the power branch (red line), the blower and driver motor are connected directly to the 24VDC line to meet their higher power requirements, especially for the immediate operation of a cooling fan when the BBS is powered on. These components receive PWM control signals from the controller via signal lines. The flow sensor is connected to the controller's signal input using its corresponding interface. Similarly, the pressure sensor is wired to the PCB mainboard, transmitting feedback data to the controller. The HMI touchscreen is then connected to the controller through both power and data lines, enabling operator interaction, configuration, and system monitoring.

**Fig. 9:**
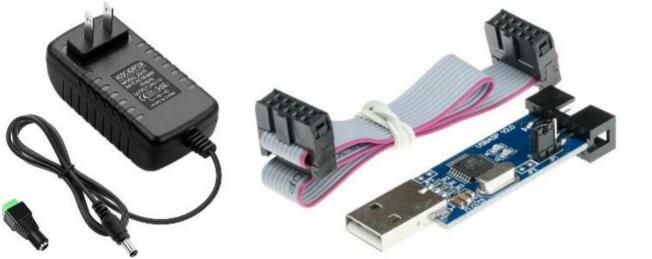
Power supply adapter and USBasp for firmware flashing.

A 24VDC adapter, as shown in Fig. 9, is used to supply the main controller and all connected components to power the BBS. For firmware flashing, a USBasp programmer is employed to upload code to the microcontroller on the control board. The USBasp is connected to the computer via USB and interfaces with the target board through a 10-pin or 6-pin in-system programming cable. Once connections are established, firmware can be compiled using tools like Atmel Studio or PlatformIO and uploaded using software such as AVRdude or eXtreme Burner. The process involves selecting the correct microcontroller, setting the appropriate fuse bits if needed, and flashing the hex file directly to the chip.

## 7 Operation instructions

**Fig. 10:**
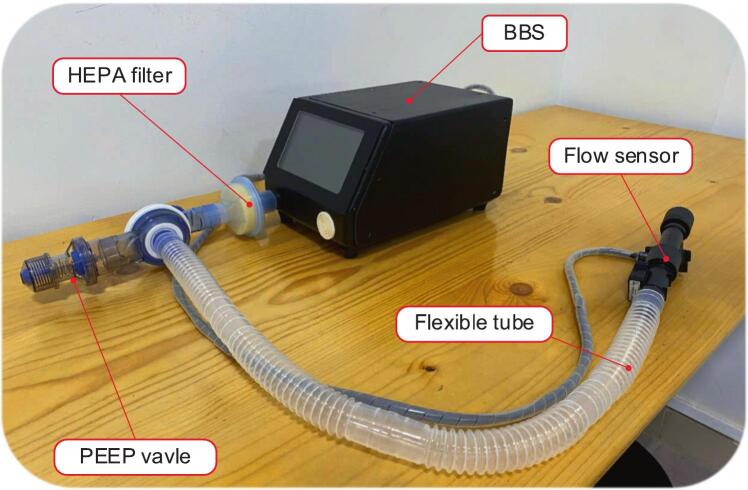
Complete assembly setup for the BBS system.

Fig. 10 shows the complete installation of the BBS system, illustrating all essential components assembled for respiratory simulation. The BBS is placed centrally and is responsible for generating controlled airflow to mimic human breathing patterns. It features a built-in HMI touchscreen for operator interaction and parameter configuration. Connected directly to the BBS is a HEPA filter, which ensures that the air output is clean and free from particulate contaminants, protecting both the BBS system and the operator. At the terminal end of the setup is a positive end-expiratory pressure (PEEP) valve, which maintains baseline airway pressure during expiration, an essential factor in simulating realistic lung mechanics and preventing airway collapse. Alongside that, between the HEPA filter and PEEP valve is a flexible tube, which transmits airflow from the BBS to the testing apparatus. Moreover, integrated within the tubing is a flow sensor, which monitors real-time airflow characteristics and provides feedback and data logging. This modular configuration allows the BBS to be easily reassembled, making it ideal for educational use, clinical research, or performance testing of respiratory equipment.

**Fig. 11:**
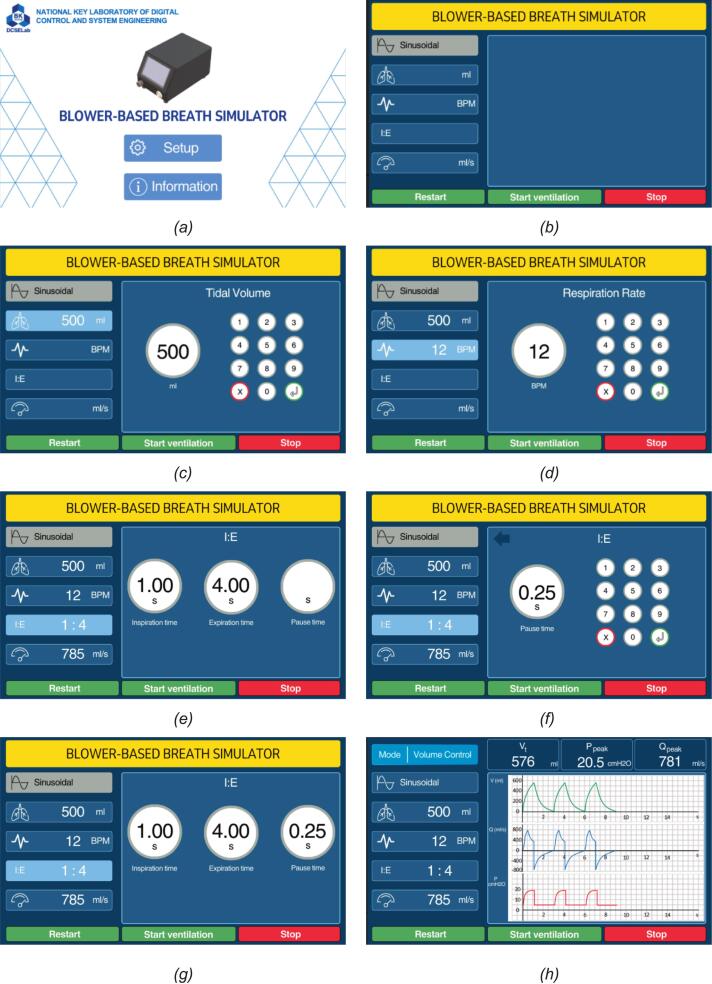
Operator interface workflow for BBS parameter configuration: (a) home screen; (b) main dashboard; (c) tidal volume setup; (d) respiration-rate setup; (e) I:E ratio adjustment; (f) pause-time setup; (g) timing configuration; (h) real-time monitoring screen.

Next, after completing the hardware assembly, the BBS system proceeds to the configuration parameters, where key ventilation parameters are set to activate the BBS. This includes defining tidal volume, respiration rate, pause time, and the inspiration-to-expiration (I:E) ratio to simulate realistic breathing patterns. All configuration steps are performed via the built-in touchscreen interface, allowing intuitive operator interaction without external control devices. The whole setup and parameter configuration process is illustrated in Fig. 11, which outlines each interface screen in detail. This ensures proper initialization of the simulator before entering the operational phase.

As shown in Fig. 11, the complete installation of the BBS illustrates the whole sequence of operator interface screens designed for configuring and monitoring the operation of a BBS. It is intended for flexible and precise simulation of human breathing patterns, with an intuitive graphical operator interface accessible via a touchscreen. Firstly, Fig. 11a is the home screen, where operators can select between ‘Setup’ to begin configuring ventilation parameters or ‘Information’ for accessing system-related documentation and operator guidance. After that, the next page is the main configuration overview as Fig. 11b. It is a summary of selected waveform types (Sinusoidal), tidal volume, respiration rate (BPM), and inspiration-to-expiration (I: E) ratio. It acts as the central dashboard before ventilation starts and provides access to modify each parameter. There, the operator sets up the first parameter, tidal volume. Fig. 11c is depicted clearly, and the setting screen allows the operator to define the desired air volume per breath (e.g., 500 ml). A virtual numeric keypad enables precise input, and the current value is shown in the center of the interface. Simultaneously, on this screen, the operator configures the Respiration Rate as in Fig. 11d, measured in breaths per minute (BPM). Like the previous screen, input is entered via a numeric keypad with the updated value displayed centrally. Fig. 11e illustrates support for I: E ratio adjustment, where operators can fine-tune the ratio of inspiration time to expiration time (e.g., 1:4). A separate field for pause time (e.g., 0.25 s) allows operators to simulate breath holding, an essential feature in replicating realistic respiratory cycles (Fig. 11f). Specifically, the Timing setup screen enables the direct configuration of inspiration time, expiration time, and pause time (e.g., 1.00, 4.00, and 0.25 s, respectively), offering more granular control over the respiratory cycle beyond fixed I: E ratios as Fig. 11g. This screen summarizes all configured parameters in a single interface for verification before operation. The tidal volume, BPM, I: E ratio, timing details, and waveform shape are all clearly presented, minimizing the risk of misconfiguration before ventilation is initiated. Finally, after completing the setup of the parameters of the BBS system, the real-time monitoring screen displays live system performance during operation, as shown in Fig. 11h. Key outputs such as measured tidal volume (492 ml), peak airway pressure (10.5 cmH_2_O), and minute ventilation (781 ml/s) are updated continuously. Two waveform graphs (pressure and flow or volume) provide visual feedback on the simulated breathing pattern. As a result, these screens offer a complete operator workflow, from setup and configuration to live monitoring, making the system suitable for research and instructional respiratory control applications.

## 8 Validation and characterization

### 8.1 Experimental setup

**Fig. 12:**
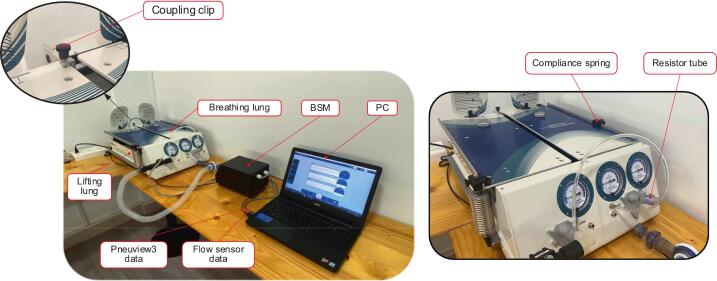
The experimental setup for the BBS validation process.

The BBS was tested to evaluate its accuracy and stability across various experimental scenarios. Fig. 12 shows the experimental setup used in this section. In this configuration, the patient’s entire biological lung system is replaced by the dual adult training test lung (TTL) from Michigan Instruments. Human spontaneous breaths are generated based on the principle of negative pressure caused by the isothermal expansion of the lungs. Therefore, to simulate such breathing using a positive-pressure device, like the one used in this study, the BBS is connected to and ventilates the left lung section of the TTL, known as the ‘lifting lung’. The lifting lung is coupled with the right lung, or the ‘breathing lung’, through a coupling clip. In this structure, the lifting lung solely simulates the diaphragm’s mechanical motion. Its compliance is set to the maximum by positioning the spring as close as possible to the rotation center of the lifting lung, without any additional resistance tube attached. Meanwhile, the breathing lung represents the patient’s lungs under various pathological conditions. Thus, this lung’s airway resistance and compliance are set to specific values for each case. Since the two lungs are connected through the coupling clip, the positive pressure generated in the lifting lung creates a lifting force that increases the volume of the breathing lung, reducing the internal pressure and drawing air inward. Based on this analysis, it can be concluded that controlling the volume of air drawn into the breathing lung can be achieved by adjusting the volume pumped into the lifting lung. Data from the two flow sensors on the BBS is transmitted to the computer via universal asynchronous receiver/transmitter communication. At the same time, measurements from the TTL are recorded using Pneuview3 software, which is integrated with this module as a reference standard for calibrating output parameters in mechanical ventilation. It should be emphasized that the TTL was employed in this study only as a validation reference and is not an integral part of the BBS system. The simulator, by virtue of its independent control and integrated display, BBS can readily be connected to other lung models or even to simple low-cost passive test systems. This flexibility confirms that BBS is intended as a standalone platform that can be adapted to diverse testing environments.

### 8.2 Experiment test scenarios

The test scenarios outlined in ISO 806601-2-79:2018 for mechanical ventilators can be applied to the BBS, as both operate on similar principles. The experiments in this section are conducted in three common lung conditions: normal lung, chronic obstructive pulmonary disease (COPD), and acute respiratory distress syndrome (ARDS). These conditions can be easily configured by using the TTL module. Unlike clinical ventilators, which can operate in both volume- and pressure-controlled modes to meet different patient needs, most breathing simulators are designed to reproduce breathing mechanics by imposing a predefined volume waveform. In these BBS systems, tidal volume is the controlled variable, while pressure arises passively from the compliance and resistance settings of the attached test lung [31], [34]. Therefore, key parameters such as tidal volume, respiration rate, inspiratory time, and PEEP need to be set. Notably, a tidal volume of 500 ml was selected as a reference value to ensure consistency across all simulated lung conditions, serving as a standardized benchmark for comparison in various scenarios rather than a clinical recommendation. The specific parameters for the three experiments are provided in Table 2.

**Table 2:** Parameter settings in three experimental scenarios.

Scenario	TTL settings		Ventilator settings			
	Lung compliance, $\frac{\mathrm{ml}}{\mathrm{cm}H_{2}O}$	Airway resistance,$\mathrm{cm}H_{2}O.\frac{s}{l}$	Tidal volume, ml	Respiration rate, bpm	Inspirational time, s	PEEP,$\mathrm{cm}H_{2}O$
Normal lung	50	5	500	20	1	5
COPD	50	20	500	12	1	5
ARDS	20	5	500	20	1	10

The breathing pacing process of the BBS goes through two phases, including inspiration and expiration. During the expiration phase, the air inside the breathing lung is expelled into the environment by the lung's elasticity. At the same time, airflow is precisely controlled during inspiration to meet specific ventilation efficiency criteria. The sliding mode controller is applied in this study to enhance tracking performance. Therefore, the first step is to evaluate the proposed controller’s effectiveness in regulating the tidal volume entering the lifting lung to follow a desired sinusoidal waveform.

**Fig. 13:**
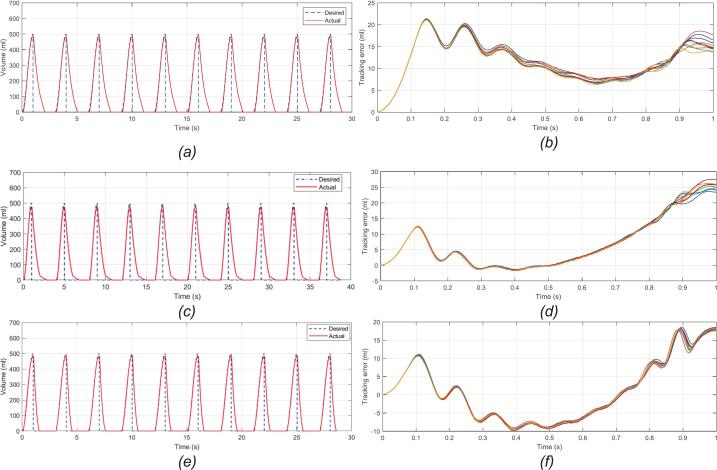
Air volume response and inspiratory tracking error, including (a) normal lung volume; (b) normal lung tracking error; (c) COPD volume; (d) COPD tracking error; (e) ARDS volume; (f) ARDS tracking error.

Figs. 13a, c, and e show the air volume response of the BBS over 10 consecutive cycles after stabilization in normal lung, COPD, and ARDS conditions, respectively, while Fig. 13, Fig. 13, Fig. 13 display the tracking error during the inspiratory cycle across these three conditions. In Fig. 13, Fig. 13, Fig. 13, it can be observed that the air volume entering the lifting lung closely follows the desired sinusoidal waveform in all three cases; however, discrepancies remain between the actual and desired air volume values, which are depicted in Fig. 13, Fig. 13, Fig. 13. Generally, in all three conditions, the tracking error increases during the first 0.1 s of inspiration, as during this period, the driver’s acceleration function gradually increases the blower speed, resulting in minimal airflow through the circuit that falls outside the sensitivity range of the flow sensor. Over the next 0.5 s, this error decreases rapidly before rising again toward the end of inspiration. During this time, the blower starts to decelerate, reducing circuit airflow and causing ‘sensor dead zone’ in the flow sensor measurements. This limitation arises when the airflow falls below the sensor’s minimum detection threshold, typically at very low flow phases such as the onset or termination of inspiration. In these regions, the sensor cannot reliably capture small airflow variations, producing zero readings. Consequently, this resolution constraint introduces tidal volume error at the end of the inspiratory cycle. Compared to the normal lung scenario, the air volume response in COPD and ARDS produces a wider error range due to external forces caused by airway resistance and lung compliance. Nevertheless, the tidal volume across all three cases maintains a small standard deviation, with a relative error below 5%, within the acceptable safety range for ventilator quality assessment. The similarity of the 10 plots in each case reflects the high repeatability and stability of the control system.

**Fig. 14:**
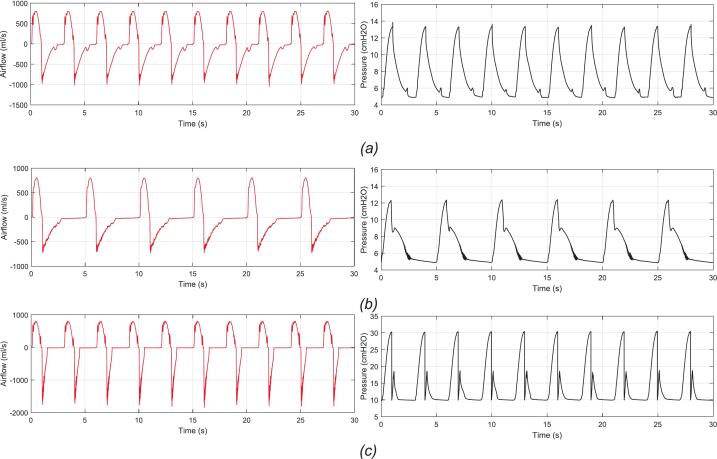
Airflow and pressure measurements in three different scenarios: (a) normal lung; (b) COPD; (c) ARDS.

Fig. 14 presents the airflow and pressure measurements for the lifting lung under three scenarios: normal lung (Fig. 14a), COPD (Fig. 14b), and ARDS (Fig. 14c). It can be observed that the volume-controlled mode consistently ensures the delivery of the set tidal volume by supplying a flow with the same amplitude, as illustrated by the red curves. However, the peak airway pressures differ, as indicated by the black curves. In the COPD case, the peak pressure is lower than that of the normal lung due to pressure loss caused by the increased resistance in the breathing lung. In contrast, the ARDS case exhibits a sharp increase in peak pressure, which is attributed to reduced lung compliance, making it more difficult for air to expand and consequently raising the airway pressure. Furthermore, thanks to the PEEP valve, positive end-expiratory pressure is maintained at the set value. Nonetheless, irregular fluctuations in pressure are observed during exhalation, caused by the spring mechanism inside the PEEP valve when the pressure approaches the set threshold. The rapid oscillation of the spring at this stage leads to transient disturbances before the system stabilizes at the equilibrium pressure. After assessing the feasibility and tracking performance of the controller in volume control (VC) mode, the BBS’s output parameters need to be compared with the actual measurements obtained from the TTL to perform calibration adjustments if necessary. According to the regulations outlined in Vietnam's measurement standard ĐLVN 331:2017, the relative error between the set parameters and actual measurements is used as a metric to evaluate the BBS’s accuracy, calculated by the following formula:

(1) $$\delta =\frac{P_{s}-P_{a}}{P_{a}}\times 100\%$$

where $P_{s}$ is the setup parameter, $P_{a}$ is the actual parameter.

**Fig. 15:**
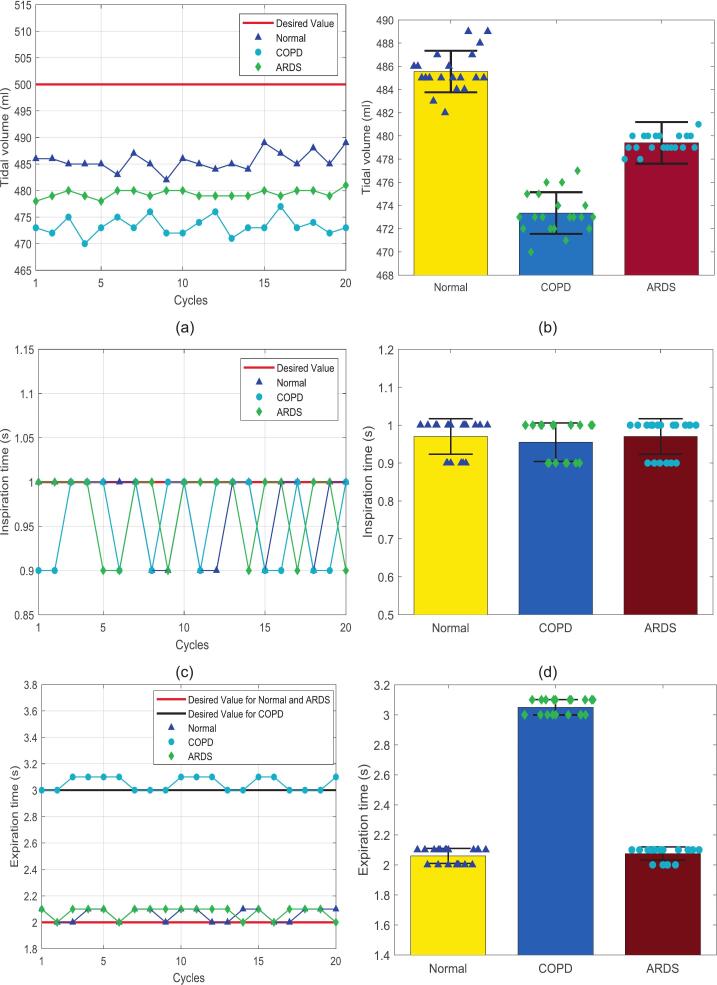
Performance of the BBS in VC mode under lung conditions different, comprising (a) tidal volume distribution in normal lung; (b) tidal volume standard deviation; (c) tidal volume distribution in COPD; (d) inspiratory/expiratory time deviation in COPD; (e) tidal volume distribution in ARDS; (f) inspiratory/expiratory time deviation in ARDS.

Three key parameters must be ensured in the operating mode of the BBS: tidal volume, inspiratory time, and expiratory time. Fig. 15, Fig. 15, Fig. 15 are scatter plots illustrating the variation of these three parameters under different lung conditions, as recorded by Pneuview3 software in 20 consecutive cycles. The measured tidal volume remained below the intended setpoint of 500  ml in all three conditions. The tidal volume hovers around 485.5 ml for normal lung conditions, slightly higher than for COPD and ARDS, but still falls short of the desired value. In the COPD condition, the tidal volume appears more stable at 473.5 ml, which is lower than in the normal lung, while in the ARDS condition, the tidal volume fluctuates around 480 ml. The highest recorded relative error was approximately 6 % in the COPD case, while the lowest was 2 % in the normal lung condition. These results suggest that the BBS encounters greater challenges in achieving the desired tidal volume in COPD and ARDS cases. This finding provides a basis for future parameter calibration steps or developing more comprehensive control algorithms. Nevertheless, the standard deviation of actual tidal volume values across all three cases is limited to a narrow range of ±2 ml, indicating the repeatability and stability of the BBS over multiple cycles (shown as Fig. 15b). Similarly, the scatter plots for inspiratory and expiratory times reveal deviations from the preset values for the BBS. Specifically, actual inspiratory times were approximately 0.1 s shorter than the desired values, while expiratory times exceeded the set value by about 0.1 s. The standard deviation of these values remained low, within ±0.1 s across all three conditions, as shown in Fig. 15, Fig. 15. This discrepancy may be attributed to the timing principle of the TTL, which initiates the inspiratory and expiratory timers upon detecting changes in airway pressure. These changes are often minimal at the beginning of a respiratory cycle and may be challenging for sensors to detect. TTL’s time resolution is limited to 0.1 s, whereas most commercial ventilators offer a time resolution to two decimal places.

**Fig. 16:**
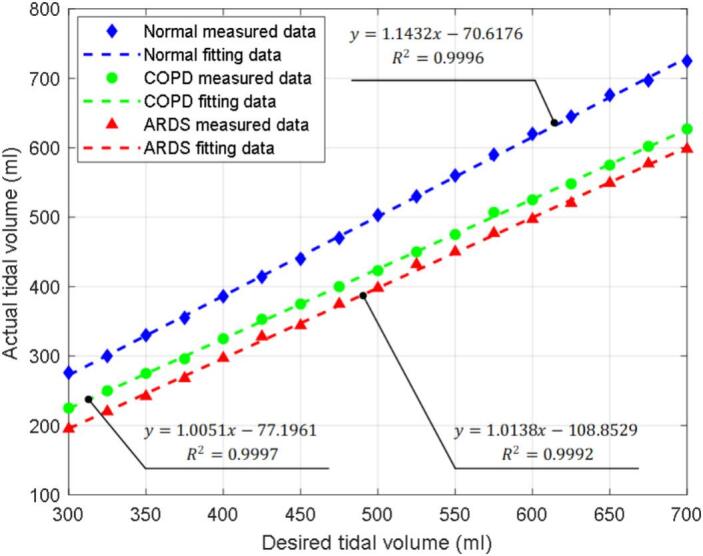
The relationship between desired and actual tidal volume in cycles. The solid points represent the experimental data, and the dotted lines represent the fitting curves by the regression model.

Fig. 16 illustrates the relationship between the desired and actual tidal volumes under three patient conditions. For each lung condition, the desired tidal volume was varied from 300 ml to 700 ml in increments of 25 ml, and the actual tidal volume was recorded using the Pneuview3 software. The regression analysis shows a clear linear dependence between the desired and measured tidal volumes, with coefficients of determination close to 1. This indicates that the BBS reproduces tidal volume changes in a consistent manner, although systematic deviations are present, as seen in the slope and offset differences across lung conditions. Such discrepancies highlight the importance of calibration, particularly for scenarios like ARDS where precise volume control is crucial. While the present study focuses on demonstrating feasibility and consistency, future work will incorporate calibration or compensation methods to minimize these errors and improve the accuracy of delivered tidal volume in clinically relevant applications.

### 8.3 Conclusion

The BBS can relatively simulate the tidal volume, inhalation, and exhalation times for both healthy individuals and those suffering from COPD and ARDS. Through experimental evaluation based on self-research and developed products, the BBS meets the following output criteria:

• Maintains input values consistently throughout the BBS’s operation.
• Demonstrates compatibility and extensibility for future in-depth research and development.
• Meets initial design criteria such as: a) lightweight, approximately 1.5 kg for the BBS and nearly 2 kg for the entire BBS system. b) low cost, around $650 per ventilator unit. c). fast production time, approximately 100 continuous hours for 3D printing and 105 h in total for the complete trial process.

In terms of drawbacks, the BBS still has an issue with its reduced accuracy under complex respiratory conditions. Additionally, the flow sensor has sensitivity thresholds that result in ‘dead zones’ during low airflow phases, affecting volume calculation. The BBS only supports volume control mode, lacks wireless data access, and remains unvalidated in clinical ventilator integration scenarios.

Future improvements for the BBS should focus on enhancing simulation accuracy and expanding usability. Auto-calibration algorithms or machine learning regression models can be integrated to correct tidal volume deviations across lung conditions. Adding support for pressure-controlled and patient-triggered modes would broaden its clinical relevance. Sensor upgrades with higher sensitivity and finer resolution will minimize dead zones. Besides, the BBS will be evaluated using a lung simulator equipped with higher-resolution temporal monitoring, allowing for more precise capture and representation of dynamic respiratory signals. Moreover, wireless connectivity and cloud-based data logging can facilitate remote monitoring and instructional use. Additionally, roadmap improvements will focus on extending compliance testing beyond ISO 80601-2-79 toward the ICU ventilator standard ISO 80601-2-12, in order to strengthen the clinical relevance of the simulator. Achieving this alignment would allow the BBS to transition from an educational and research platform into a potential candidate for emergency or critical-care applications. Finally, clinical validation with real ventilators could elevate the BBS to a higher certification-ready standard.

## CRediT authorship contribution statement

**Cong Toai Truong:** Writing – original draft, Resources, Methodology, Investigation, Conceptualization. **Trung Dat Phan:** Visualization, Software, Data curation, Conceptualization. **Ly Xuan Truong Pham:** Software, Investigation, Data curation. **Huy Hung Nguyen:** Validation, Project administration, Formal analysis. **Tan Tien Nguyen:** Supervision, Funding acquisition. **Van Tu Duong:** Writing – review & editing, Methodology, Conceptualization.

## Declaration of competing interest

The authors declare that they have no known competing financial interests or personal relationships that could have appeared to influence the work reported in this paper.
