# Supplementary material for: Design of a low-cost, portable blower-based breath simulator using 3D printing for respiratory research and education
Source: HardwareX. 2025 Dec 14;25:e00731. doi: 10.1016/j.ohx.2025.e00731 (PMC12808584; doi:10.1016/j.ohx.2025.e00731)
Supplement: Supplementary Data 2 [file mmc2.zip › BVM_Main/BSM_Main.PcbDoc.htm]

Reporting Options

# File in Newer Format

|  |  |  |
| --- | --- | --- |
| Date | : | 5/13/2025 |
| Time | : | 12:43:29 PM |
| Filename | : | C:\Users\truon\Desktop\BVM\_Main\BSM\_Main.PcbDoc |

  

| Version | Warning |
| --- | --- |
| Release 20.0 | **CAUTION** - Support of propagation delay for primitives |

  

---

This file was generated by **a later** version of the software
