# Supplementary figures and images for: Design of a low-cost, portable blower-based breath simulator using 3D printing for respiratory research and education
Source: HardwareX. 2025 Dec 14;25:e00731. doi: 10.1016/j.ohx.2025.e00731 (PMC12808584; doi:10.1016/j.ohx.2025.e00731)

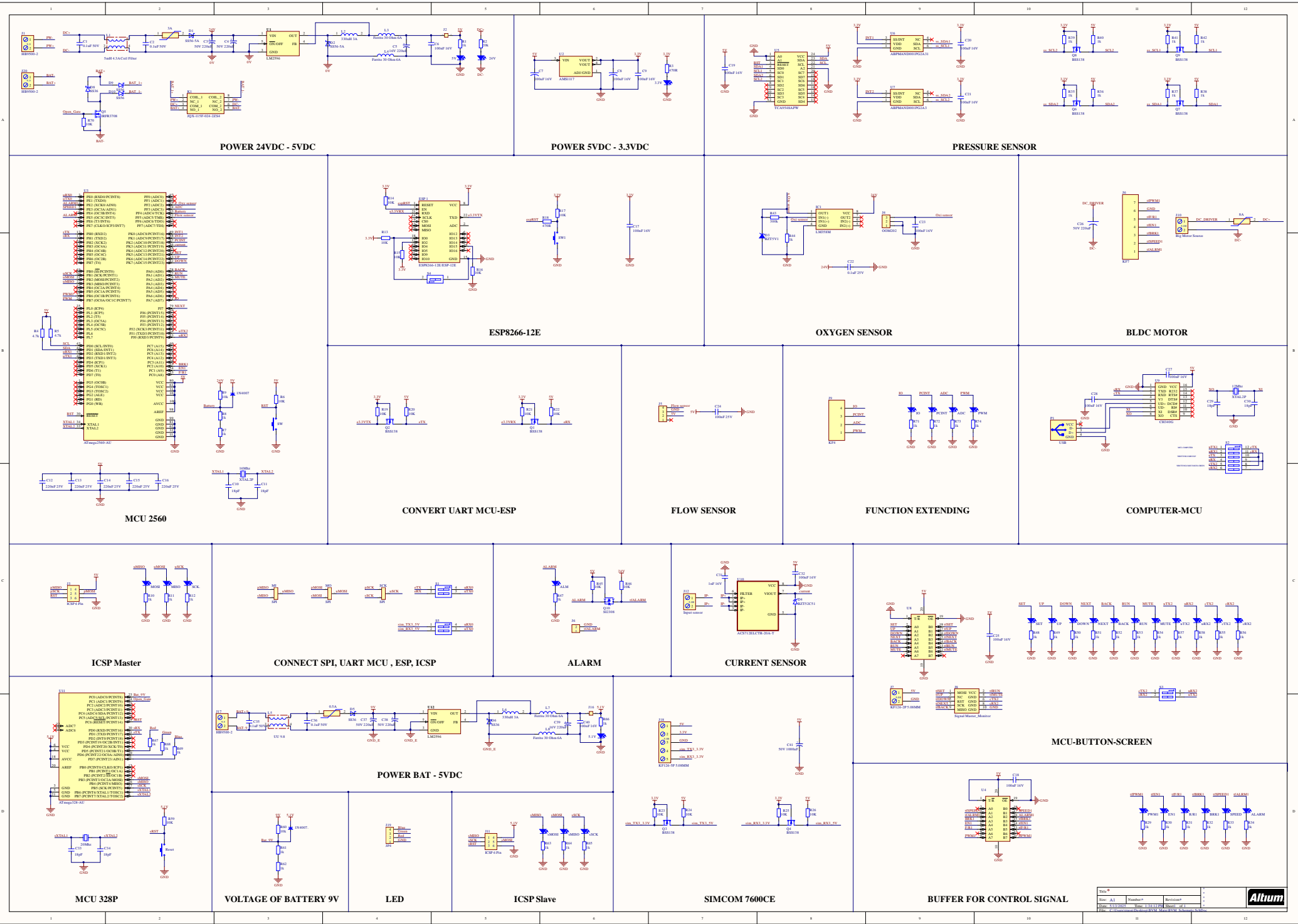

Supplement: Supplementary Data 2 [file mmc2.zip › BVM_Main/BSM_Project.pdf]
