# Supplementary material for: Design of a low-cost, portable blower-based breath simulator using 3D printing for respiratory research and education
Source: HardwareX. 2025 Dec 14;25:e00731. doi: 10.1016/j.ohx.2025.e00731 (PMC12808584; doi:10.1016/j.ohx.2025.e00731)
Supplement: Supplementary Data 3 [file mmc3.pdf]

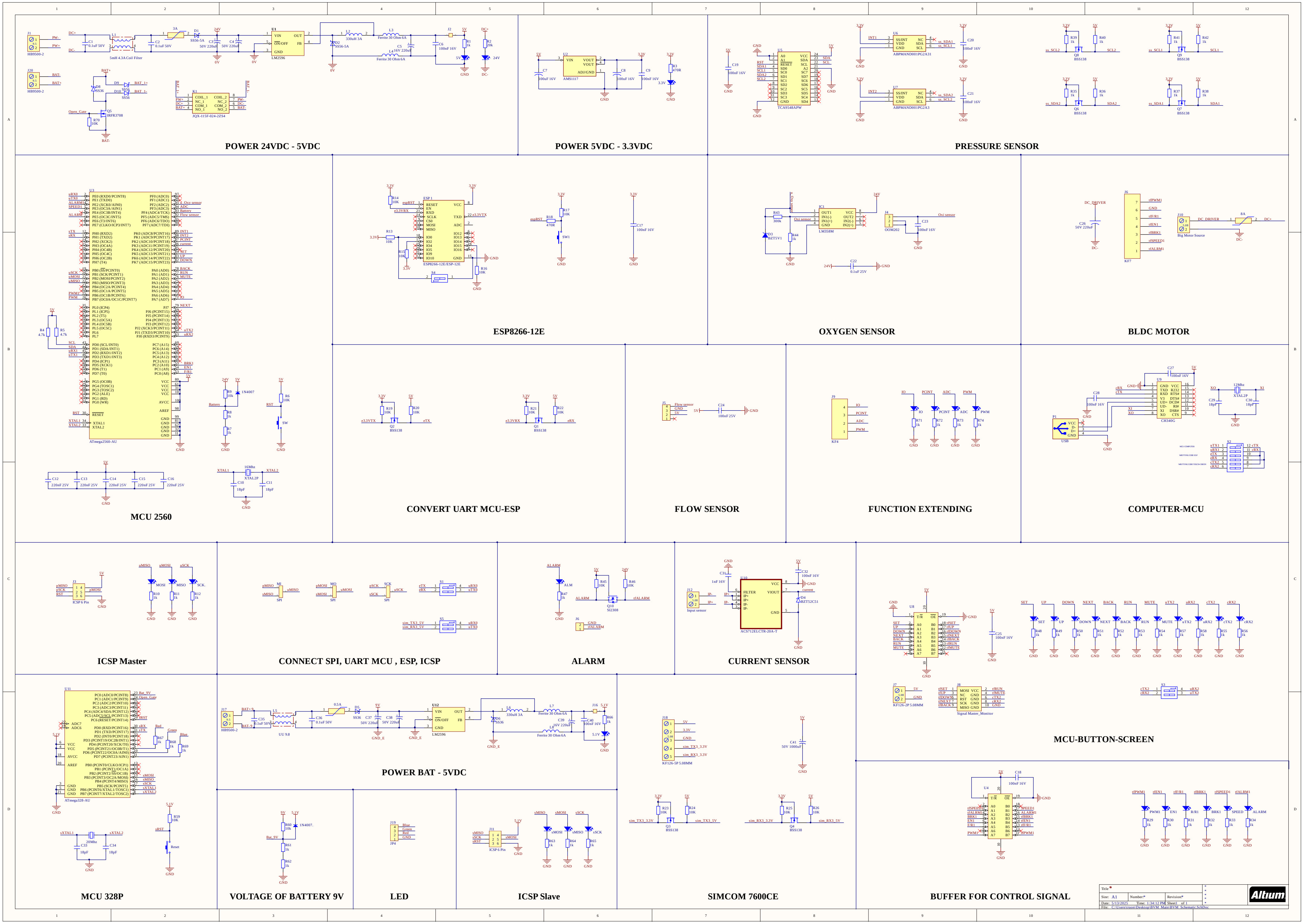
POWER 24VDC - 5VDC
POWER 5VDC - 3.3VDC
PRESSURE SENSOR
MCU 2560
ESP8266-12E
OXYGEN SENSOR
BLDC MOTOR
CONVERT UART MCU-ESP
FLOW SENSOR
FUNCTION EXTENDING
COMPUTER-MCU
ICSP Master
CONNECT SPI, UART MCU , ESP, ICSP
ALARM
CURRENT SENSOR
MCU-BUTTON-SCREEN
POWER BAT - 5VDC
MCU 328P
VOLTAGE OF BATTERY 9V
LED
ICSP Slave
SIMCOM 7600CE
BUFFER FOR CONTROL SIGNAL
Title *
Size: A1
Number:*
Revision:*
Date: 5/13/2025
Time: 1:34:12 PM
Sheet1 of 1
File: C:\Users\truon\Desktop\BVM_Main\BVM_Schematic.SchDoc
